# Distinct patterns of osteoradionecrosis after photon-based radiotherapy and carbon ion radiotherapy for unresectable adenoid cystic carcinoma of the head and neck: case series from two institutions

**DOI:** 10.2340/1651-226X.2025.42209

**Published:** 2025-01-15

**Authors:** Eivind Storaas, Marta D. Switlyk, Sigrun Dahl, Cecilie D. Amdal, Åse Bratland, Thuy-Tien M. Huynh, Hanne A. Eide, Barbara Vischioni, Ester Orlandi, Einar Dale

**Affiliations:** a Department of Oncology, Oslo University Hospital, Oslo, Norway; b Department of Radiology and Nuclear Medicine, Oslo University Hospital, Oslo, Norway; c Faculty of Medicine, University of Oslo, Oslo, Norway; d Clinical Department, National Center for Oncological Hadrontherapy (Fondazione CNAO), Pavia, Italy; e Department of Clinical, Surgical, Diagnostic, and Pediatric Sciences, University of Pavia, Pavia, Italy

**Keywords:** Adenoid cystic carcinoma of the head and neck, carbon-ion radiotherapy, osteoradionecrosis

## Abstract

**Background and purpose:**

To present the clinical outcomes of two series of patients treated with carbon-ion radiotherapy (CIRT) and definitive photon radiotherapy (RT) for adenoid cystic carcinoma of the head and neck (HN-ACC).

**Material and methods:**

The first cohort of six patients was referred from Oslo University Hospital (OUS) to Centro Nazionale di Adroterapia Oncologica (CNAO, Pavia, Italy) for CIRT in 2014–2017. The second cohort included 18 patients treated with definitive photon RT at OUS in 2005–2017. The primary endpoint was an evaluation of osteoradionecrosis (ORN) in the two cohorts. The secondary endpoints were treatment efficacy by local control (LC), progression-free survival (PFS), and overall survival (OS).

**Results:**

The tumor stage was T4 for all the patients in the CIRT group and 15 (84%) in the photon group. There were three (50%) patients with grade 3 ORN in the CIRT group compared to one (6%) with grade 3 ORN in the photon group (*p* = 0.05). The 5-year LC (95% CI), PFS, and OS rates in the CIRT group and the photon group were 33% (11–100) and 39% (19–76), 17% (9–100) and 23% (2–59), and 80% (52–100) and 50% (31–82), respectively.

**Interpretation:**

Half of the patients in the CIRT cohort experienced ORN requiring surgical management during the follow-up. Patients with ACC referred for CIRT often have a worse prognosis and more advanced disease than patients treated with photons. When returning from the referring center, these patients need close follow-up often in collaboration with treating centers to manage toxicity that impacts quality of life.

## Introduction

Salivary gland malignancies account for up to 5% of head and neck cancers [1]. Adenoid cystic carcinoma (HN-ACC), a subset of these, is typically slow growing but locally aggressive, often spreading along skull base nerves. Advanced cases may involve vital structures, making surgery unfeasible. Standard treatment for localized disease combines surgery and postoperative photon radiotherapy (RT), achieving 5-year local control (LC) rates of 68–91% [2]. However, unresectable cases treated with photon RT show poor outcomes [3]. Due to its rarity and diagnostic challenges, especially in high-grade cases, clinical research on HN-ACC is limited, and consensus on the management is slowly emerging [4]. In this regard, patients with an expected R2 margin are preferably sent for primary RT and heavy ion therapy [5].

Carbon-ion radiotherapy (CIRT) is emerging as a promising option for inoperable HN-ACC, offering high linear energy transfer (LET) and precise spatial distribution through the Bragg peak. These properties may overcome the radioresistance and hypoxia of HN-ACC [6]. Recent studies suggest that CIRT improves LC, overall survival (OS), and toxicity outcomes, even in R2-resected or inoperable cases [7, 8].

Osteoradionecrosis (ORN), a painful condition from radiation-induced tissue injury, arises when hypoxic, hypovascular, and hypocellular tissue fails to heal [9]. Spontaneous ORN correlates with radiation dose and significantly impacts quality of life [10, 11]. Treatment ranges from improved oral hygiene and antibiotics to surgery and hyperbaric oxygen therapy. Severe ORN (grade ≥ 3) rates are 2–4% with modern photon RT [12, 13] and 4–21% after CIRT [14, 15].

Since 2014, Oslo University Hospital (OUS) has referred younger, inoperable HN-ACC patients for primary CIRT at Centro Nazionale di Adroterapia Oncologica (CNAO) in Italy, which has treated over 300 HN-ACC cases since 2010. This study aimed to report ORN frequency and survival outcomes for Norwegian patients treated with CIRT at CNAO, compared with photon RT at OUS.

## Material and methods

This is a retrospective observational study of patients with unresectable HN-ACC who received treatment from 2005 to 2017. There were two groups of patients: the CIRT group treated at CNAO and the photon group treated at OUS.

The patients in the CIRT group (*n* = 6) were referred to CNAO from 2014 to 2017. These patients received RT with carbon ions to a total dose of 65.6–68.8 Gy (relative biological effectiveness [RBE]) in 4.1–4.3 Gy per fraction (RBE), as described by Vischioni et al. [16].

The patients in the photon group (*n* = 18) were treated at OUS with primary RT using photons. Planning computed tomography (CT) was performed on an RT-compatible flat table with head support and a fixation mask. Most of the patients were treated with two sequential plans. First, 46 Gy in 2 Gy per fraction to the gross tumor volume (GTV) plus 10 mm margin to the clinical target volume (CTV) including the elective neck volume, followed by 24 Gy in 2 Gy per fraction to the GTV. The planning target volume (PTV) margin to the CTV was 3 mm (Supplementary Table 1).

A complete dental examination was requested prior to treatment in both groups. If clinically indicated, tooth extraction was performed at least 14 days before the start of treatment.

Patient and tumor characteristics were collected from the medical records: age, sex, performance status (Eastern Cooperative Oncology Group [ECOG] status), comorbidity [17], tobacco use, tumor site, TNM stage, and follow-up information. TNM classification was reassessed according to the American Joint Committee on Cancer (AJCC)/Union for International Cancer Control (UICC) 8th edition. The number of teeth in the PTV was recorded as this parameter has been shown to correlate with the risk of ORN [16].

The patients were followed every 3 months for at least 2 years, thereafter every 6 months for 5 years or more. The institutional practice was to perform imaging (magnetic resonance imaging or CT) 3 months after RT and then as clinically indicated. Grade 2 and 3 ORNs were scored with the available imaging and patient data according to the Common Terminology Criteria for Adverse Events (CTCAE) v5.0 [18]. Follow-up in this study was terminated on December 6, 2023.

### Statistics

Frequencies in contingency tables were compared using Fisher’s exact test. For calculation of survival, the Kaplan–Meier method was used. Time was measured from the start of RT to an event or censoring. Progression-free survival (PFS), LC, regional, and distant failure were counted either by biopsy or by radiological findings if biopsy was not warranted. The median follow-up time was estimated using the reverse Kaplan–Meier method. *P*-values <0.05 were considered statistically significant. The data were analyzed using RStudio version 2022.2.1.461 with R version 4.1.2 integrated [19].

## Results

Patient characteristics of the CIRT group (*n* = 6) and the photon group (*n* = 18) are presented in Table 1. The six patients treated with CIRT were younger (*p* = 0.001), had larger tumors (*p* = 0.05), and more often had skull base infiltrating tumors (*p* = 0.01) compared to the 18 patients treated with photons. There was a tendency toward more bone infiltrating tumors in the CIRT group (*p* = 0.06).

**Table 1:** Patient and tumor characteristics.

Characteristic	Photons (*n* = 18) *n* (%)	Carbon ions (*n* = 6) *n* (%)	*P*
Age (years), median (range)	67 (38, 81)	41 (31, 49)	**0.001**
Sex			
Male	8 (44)	2 (33)	1
Female	10 (56)	4 (67)	
ECOG performance status ≥ 1	3 (17)	2 (33)	0.27
Charlson comorbidity index ≥ 1	7 (39)	1 (17)	0.62
Smoker	10 (56)	2 (33)	0.64
Tumor site			
Sinonasal/nasopharynx	2 (11)	5 (83)	**0.003**
Oropharynx	6 (33)	0 (0)	0.27
Oral cavity	4 (22)	0 (0)	0.54
Major salivary glands	5 (28)	1 (17)	1
Lacrimal gland	1 (6)	0 (0)	1
Skull base involvement	4 (22)	5 (83)	**0.01**
Bone infiltration	6 (33)	5 (83)	**0.06**
Tumor classification			
T1	0 (0)	0 (0)	1
T2	2 (11)	0 (0)	1
T3	1 (5)	0 (0)	1
T4	15 (84)	6 (100)	1
Nodal staging			
N0	15 (83)	6 (100)	0.55
N+	3 (17)	0 (0)	
M0	16 (89)	6 (100)	1
M1	2 (11)	0 (0)	
GTV (cm^3^), median (range)	18 (2, 214)	64 (34, 114)	**0.05**

ECOG: Eastern Cooperative Oncology Group; GTV: gross tumor volume.

In the CIRT group, there were three incidents of grade 3 ORN and one in the photon group (Table 2). CIRT patient no. 4 had a parotid gland tumor without bone infiltration and later developed extensive ORN in the temporal bone starting from otitis in the external auditory canal. This patient needed surgical revision of the ORN. Eighteen months prior to the onset of ORN, the multityrosine kinase inhibitor lenvatinib was started because of local recurrence and lung metastases. The medication was discontinued when the ORN was verified. Two other patients in the CIRT group and two patients in the photon group also used lenvatinib without the occurrence of ORN.

**Table 2:** Details on patients who received carbon-ion radiotherapy (CIRT) and photons and the occurrence of grade 3 osteoradionecrosis (ORN).

Patient no.	RT modality	Age (years)	Sex	Tumor location	TNM	Bone infiltration	ORN grade 3	Time of diagnosis/surgery ORN (months after RT)	Number of teeth covered by high dose^1^	Overall survival (months after RT)
1	CIRT	49	F	Sinonasal	T4aN0M0	Yes	÷	÷	6	52
2	CIRT	45	F	Sinonasal	T4bN0M0	Yes	÷	÷	7	68
3^2^	CIRT	49	M	Nasopharynx	T4aN0M0	Yes	Mandible	34/35	1	Alive (85)
4	CIRT	41	M	Parotid	T4aN0M0	No	Temporal	46/46	0	81
5^3^	CIRT	31	F	Sinonasal	T4bN0M0	Yes	Mandible	18/45	7	Alive (81)
6	CIRT	36	F	Sinonasal	T4bN0M0	Yes	÷	÷	0	Alive (75)
1	Photons	81	M	Sinonasal	T4bN0M0	No	÷	÷	0^4^	2
2	Photons	61	M	Oropharynx	T3N1M0	No	÷	÷	2	26
3	Photons	78	F	Parotid	T4aN0M0	Yes	÷	÷	0	2
4	Photons	76	M	Parotid	T4aN0M0	Yes	÷	÷	0^4^	50
5	Photons	56	F	Oropharynx	T4aN0M0	No	÷	÷	2	110
6	Photons	63	F	Oral cavity	T4aN0M0	Yes	÷	÷	5	111
7	Photons	38	F	Oral cavity	T4aN0M0	Yes	÷	÷	2	63
8	Photons	70	F	Oropharynx	T4aN0M0	No	÷	÷	0^4^	Alive (136)
9	Photons	71	F	Sinonasal	T4bN0M0	Yes	÷	÷	0^4^	17
10	Photons	54	M	Oral cavity	T2N1M1	Yes	Maxilla	72	8	Alive (125)
11	Photons	74	M	Oral cavity	T4bN0M0	No	÷	÷	0^4^	Alive (121)
12	Photons	53	M	Parotid	T4aN0M0	No	÷	÷	0	105
13	Photons	66	F	Parotid	T4aN0M0	No	÷	÷	0	Alive (105)
14	Photons	68	F	Lacrimal duct	T4bN0M1	No	÷	÷	0	28
15	Photons	67	F	Oropharynx	T4aN2bM0	No	÷	÷	7	41
16	Photons	64	M	Oropharynx	T4aN0M0	No	÷	÷	18	70
17	Photons	76	M	Parotid	T2N0M0	No	÷	÷	0	52
18	Photons	72	F	Oropharynx	T4aN0M0	No	÷	÷	0	Alive (113)

RT: radiotherapy.

Two rows are shaded because of very short overall survival.

1 Definition of ‘High dose’: For CIRT, the number of teeth in the planning target volume (PTV). For photons, the number of teeth covered by 60 Gy isodose.

2 CIRT patient no. 3 was also diagnosed with grade 2 ORN in the maxilla 36 months after CIRT.

3 CIRT patient no. 5 was also diagnosed with grade 2 ORN in the maxilla 77 months after CIRT.

4 Jaw(s) covered by a high dose, but edentulous before radiotherapy (RT).

**Figure 1A–D:**
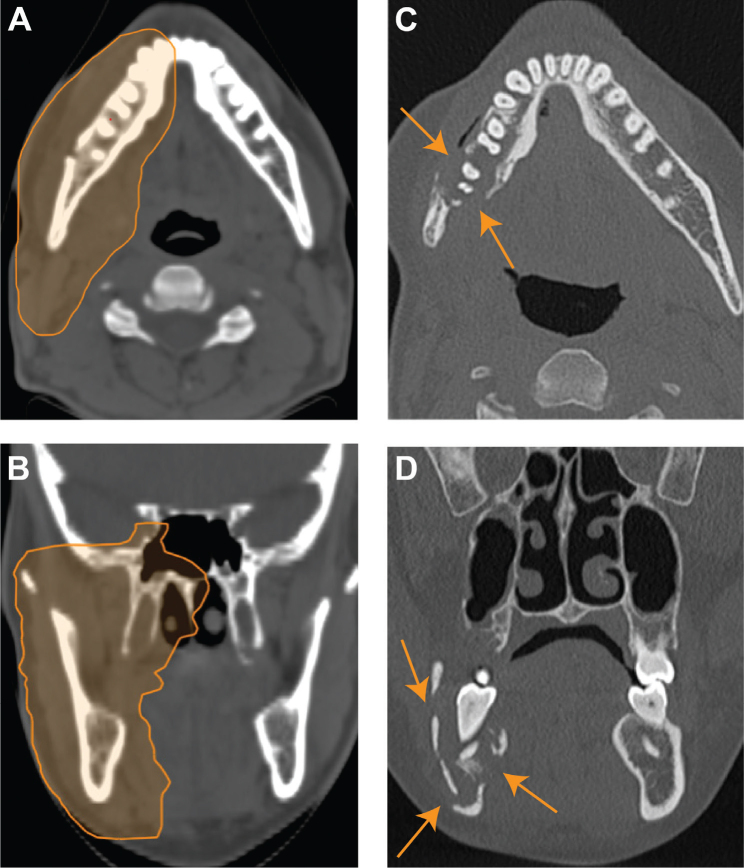
Carbon-ion radiotherapy (CIRT) patient no. 5: Osteoradionecrosis (ORN) development: Axial (A) and coronal (B) views of the treatment plan with the planning target volume (PTV) prescribed 68.8 Gy (relative biological effectiveness [RBE]). Axial (C) and coronal (D) computed tomography (CT) scans show a lucent area with trabecular loss, bicortical erosion, and pathological fracture (arrows). The findings are consistent with grade 3 ORN involving the right mandibular body and angle, which was diagnosed 18 months after CIRT.

The other two CIRT patients with ORN experienced affection of the mandible. One of them, CIRT patient no. 5, had a paranasal sinus tumor with growth along the mandibular nerve, involvement of the mandible, and seven teeth in the PTV (Table 2 and Figure 1). This patient was later diagnosed with an additional grade 2 ORN in the maxilla. CIRT patient no. 3 did not have tumor involving the mandible but experienced ORN related to a tooth extraction (molar) 6 months before the onset of ORN. The tooth was extracted because of serious caries despite that the teeth were in healthy condition before CIRT. This patient also developed grade 2 maxillary ORN following another tooth extraction. Patient nos. 3 and 5 with mandibular ORN needed a hemi-mandibulectomy. In the photon group, one patient with tumor infiltration of the hard palate experienced grade 3 ORN of the maxilla and needed surgical revision. The difference in the number of grade 3 ORNs across the groups was statistically significant (*p* = 0.05). There was no grade 2 ORN in the photon group. All the grade 3 ORNs in both groups were within the PTV. Only one of the patients with ORN (CIRT patient no. 3) had a history of smoking.

The median follow-up time in the CIRT group was 83 months (range 52–85 months) and 121 months (range 2–136 months) in the photon group. The 5-year LC rates in the CIRT group and the photon group were 33% (95% confidence interval: 11–100%) and 39% (19–76%), respectively. The 5-year PFS rates in the CIRT group and the photon group were 17% (9–100%) and 23% (2–59%), respectively. The OS after 5 years was 80% (52–100%) in the CIRT group and 50% (31–82%) in the photon group.

In the CIRT group, there were four cases of local recurrence. All recurrences were within the PTV except for one patient who experienced local recurrence at the border of the PTV. None of the CIRT patients had metastatic lymph nodes in the neck at the time of diagnosis. One patient developed a regional recurrence 51 months after treatment. The same patient experienced a local recurrence 30 months earlier. None of the CIRT patients received elective neck irradiation.

In the photon RT group, two patients aged 78 and 81 years died of unknown causes only 2 months after RT. Of the remaining 16 patients, 12 had local disease recurrence. All recurrences were in the PTV except for one patient with a parotid gland tumor who experienced perineural recurrence in the skull base at the border of the PTV.

Three patients in the photon group had metastatic lymph nodes in the neck at the time of diagnosis. One of them underwent elective neck dissection following RT with viable tumor tissue in the specimen, with no sign of recurrence thereafter. The other two patients achieved regional control of the disease in the high-dose area. One of these two patients had both local disease recurrence (in the high-dose area) and regional disease recurrence at the border of the elective neck volume 69 months after RT. This was the only patient experiencing regional recurrence of the disease in the photon group. Eleven patients received elective neck irradiation.

## Discussion

This study assessed outcomes for Norwegian patients with HN-ACC treated with CIRT at CNAO and photon RT at OUS. In the CIRT group, three of six patients (50%) experienced grade 3 ORN, a higher rate than in prior studies. For example, Koto et al. reported a 4% grade 3 ORN rate among 458 patients with sinonasal malignancies treated with CIRT, likely due to lower mandibular doses in their cohort [20]. Mandibular ORN risk is typically higher than maxillary ORN [21], but the risk is also influenced by tumor site, treatment volume, and high-dose exposure. This seems to be confirmed by Ikawa et al. who found a 12% ORN rate in nonsquamous cell oral cavity cancers [22], while Naganawa et al. reported a 21% rate in oral mucosal melanoma cases [15]. By contrast, photon RT studies report lower ORN rates of 2–4% [12, 13]. Notably, previous CIRT studies excluded cases where ORN was likely linked to tumor bone infiltration since ORN might be an expected result of tumor response [16, 23, 24], whereas such patients were included here.

Dental factors contributed to ORN in two CIRT cases, emphasizing the need for individualized dental extraction protocols. One patient’s ORN may also have been exacerbated by lenvatinib, a VEGF inhibitor linked to impaired bone healing [25–27]. VEGF is crucial for bone repair, and the jaw’s high bone turnover makes it particularly vulnerable to disruptions in osteoblast and osteoclast function. Future studies should clarify the effects of combining CIRT with VEGF inhibitors and optimize treatment timing for systemic disease progression.

CIRT is normally administered at a higher dose per fraction than conventional photon or proton RT, with doses equivalent to 100 Gy (EQD2Gy) [28] exceeding standard photon RT dose constraints for bone [29, 30]. This high dose, combined with advanced disease features, likely contributed to the observed ORN rates.

The 5-year LC rate in the CIRT group was 33%, which is lower than the previously reported rates of 68% in Japanese centers [7], 59% in Heidelberg using a carbon-ion boost [8], and 52% reported by CNAO [31]. Factors such as skull base involvement, bone infiltration, and large tumors likely reduced LC in the present cohort [32–34].

CIRT has emerged as a potential treatment for inoperable HN-ACC, offering a chance of local tumor control for patients with advanced disease. However, the small sample size and retrospective design of this study limit its generalizability. Toxicity grading relied on available data, and differences in patient and tumor characteristics between the CIRT and photon cohorts complicated direct comparisons. Patients referred for CIRT typically presented with more unfavorable disease features, such as larger tumors and skull base involvement, introducing selection bias.

In conclusion, half of the CIRT cohort experienced ORN requiring surgery during follow-up at their home institution. Younger patients with advanced disease and poorer prognoses are often referred to particle therapy centers for CIRT. Close follow-up in collaboration with these centers is crucial to manage treatment-related toxicity. Careful patient selection and thorough risk communication are essential. Despite the limitations of this small, retrospective study, these findings may be of value to other centers when referring patients with inoperable HN-ACC for CIRT abroad.

### Statement of ethics

The study was approved by The Regional Ethics Committee (REK) in Norway (approval number 198926) and the Institutional Review Board at OUS. Informed consent was obtained from all living patients. An exemption from obtaining informed consent from the relatives of deceased patients was granted by REK.

## Supplementary Material

Distinct patterns of osteoradionecrosis after photon-based radiotherapy and carbon ion radiotherapy for unresectable adenoid cystic carcinoma of the head and neck: case series from two institutions

## Data Availability

Data will be made available upon reasonable request.
