# Supplementary material for: Distinct patterns of osteoradionecrosis after photon-based radiotherapy and carbon ion radiotherapy for unresectable adenoid cystic carcinoma of the head and neck: case series from two institutions
Source: Acta Oncol. 2025 Jan 15;64:42209. doi: 10.2340/1651-226X.2025.42209 (PMC11758679; doi:10.2340/1651-226X.2025.42209)
Supplement: Distinct patterns of osteoradionecrosis after photon-based radiotherapy and carbon ion radiotherapy for unresectable adenoid cystic carcinoma of the head and neck: case series from two institutions [file AO-64-42209-s1.pdf]

Supplementary material has been published as submitted. It has not been copyedited, or typeset by Acta Oncologica

Supplementary Table 1: Characteristics of radiotherapy. For carbon-ion radiotherapy, the relative biological effectiveness (RBE)-weighted dose is shown.

	Photons	Carbon ions
Dose (Gy) - median (range)	70 (66 - 70)	68.8 (66 - 69)
Dose per fraction (Gy) - median (range)	2 (2 - 2)	4.3 (4.1 - 4.3)
Modulated RT - n (%)	12 (67%)	6 (100%)
Elective neck RT - n (%)	11 (61%)	0 (0%)
